# Biochemical Atypia in Russian *Neisseria gonorrhoeae* Clinical Isolates Belonging to the G807 NG-MAST Genogroup/ST1594 MLST

**DOI:** 10.3390/microorganisms10112271

**Published:** 2022-11-16

**Authors:** Nikita Nosov, Alexey Kubanov, Viktoria Solomka, Dmitry Deryabin

**Affiliations:** State Research Center of Dermatovenerology and Cosmetology of Russian Ministry of Health, Korolenko Street, 3, 107076 Moscow, Russia

**Keywords:** *Neisseria gonorrhoeae*, biochemical atypia, phylogenetic analysis

## Abstract

Many current gonococcal clinical isolates in Russia show atypical taxonomically significant biochemical activity, which leads to species misidentification. Molecular typing of such cultures according *Neisseria gonorrhoeae* multiantigen sequence typing (NG-MAST) and multilocus sequence typing (MLST) protocols assigned them to the G807 NG-MAST GENOGROUP/ST1594 MLST that has been predominant in Russia in recent years. The goal of the study was to analyze the molecular mechanisms of biochemical atypia in *N. gonorrhoeae* clinical isolates characterized as the members of G807 NG-MAST GENOGROUP/ST1594 MLST. Sixteen isolates of this genogroup were included in the study, eight showed defective amino acid metabolism or loss of D-glucose fermentation. Comparative bioinformatic analysis based on WGS data divided these isolates into two clusters strictly associated with typical or atypical biochemical activity. Cultures with defective amino acid metabolism had a 5-nucleotide insertion in the *pip*-gene that caused a stop codon and led to synthesis of the non-functional enzyme. Comparison of the sequenced genomes with publicly available *N. gonorrhoeae* genomes showed the rarity of this insertion. In the global *N. gonorrhoeae* phylogenetic tree the G807 NG-MAST GENOGROUP/ST1594 MLST forms a distinct branch characterized by 170 SNPs, most of which are non-synonymous. We hypothesized a unique strategy for G807 NG-MAST GENOGROUP/ST1594 MLST clone persistence in the global *N. gonorrhoeae* population via escape of antimicrobial therapy due to diagnostic misidentification.

## 1. Introduction

Gonorrhea is one of the most common STIs worldwide. Despite the general trend toward a decrease in the relative incidence of gonorrhea, the reproductive health risks associated with the disease remain, such as reduced fertility, pelvic inflammation, ectopic pregnancy, and others. The constant growth of resistance of the gonorrhea pathogen, *Neisseria gonorrhoeae*, to antibacterial drugs used in therapy creates the risk of incurable forms of gonococcal infection [1]. In this regard, the WHO in 2017 included *N. gonorrhoeae* in the list of 12 bacterial pathogens for which the development of new antibiotics is urgently required [2].

Despite the widespread use of nucleic acid amplification-based methods for diagnosing gonococcal infection, culture remains the gold standard; it allows not only identification of the pathogen, but also assessment of its sensitivity to antimicrobial agents, and additional studies, including biochemical activity study. The Russian guideline, in accordance with WHO recommendations, uses PCR for diagnosis of gonorrhea, and therapy is possible after full identification of the microorganism following culture diagnosis and microbiological identification.

In accordance with the protocols of the 2004 WHO-initiated Gonococcal Antimicrobial Surveillance Programme (GASP), the Russian version of this program for monitoring gonococcal drug sensitivity, recognized in the international scientific community as RU-GASP, was launched at the State Scientific Center of Dermatovenerology and Cosmetology of the Russian Ministry of Health. The program analyzes clinical isolates of *N. gonorrhoeae* obtained from dermatovenerological medical institutions of 40 subjects of the Russian Federation. This program has been carried out continuously for 18 years and its results are regularly published in the scientific and reporting documents of WHO on this problem. The key steps in cultural testing are isolation and identification of the pathogen, traditionally based on a combination of biochemical activity tests (including automated analyzers). However, when conducting such studies as part of the RU-GASP program, we encountered the fact that a significant number of clinical isolates obtained in the Russian Federation since 2016 showed signs of biochemical atypia and were incorrectly identified based on the results of triplicate biochemical activity tests. The atypia was associated with a loss of activity in four enzymes of amino acid metabolism: L-prolarylamidase (ProA), L-alanylphenylanylprolarylamidase (APPA), tyrosinarilamidase (TyrA), argininearylamidase (ArgA), and D-glucose (dGlu) fermentation ability [3]. At the same time, analysis of these cultures on a MALDI Microflex time-of-flight ionization mass spectrometer (Bruker Daltonics GmbH, Germany) clearly assigned them to the *N. gonorrhoeae* species with an identification index above 2.0.

Further molecular typing of such clinical isolates using the *N. gonorrhoeae* multiantigen sequence typing (NG-MAST) protocol assigned them to genogroup G807 NG-MAST, which has been one of the dominant types in the Russian Federation in recent years [4], or to genetically related sequencing types distinguished by single nucleotide substitutions in the *porB* or *tbpB* genes. ST807 and related sequence types are included in the large G51 genogroup [5]. In turn, NG-MLST typing assigned them to a single MLST 1594 sequencing type, which also confirmed their common origin.

The aim of this study was to compare the genomes of Russian clinical isolates belonging to the G807 NG-MAST GENOGROUP/ST1594 MLST to find the causes of biochemical atypia and to determine their phylogenetic position in the global *N. gonorrhoeae* population.

## 2. Materials and Methods

### 2.1. Specimens (Clinical Isolates) and N. gonorrhoeae Genome Information

NG-MAST typing was performed by analyzing the nucleotide sequences of *porB* (490 bp) and *tbpB* (390 bp) variable gene sites according to a known protocol [6] using the database at the internet resource PubMLST and Ugene 41 software. MLST typing was performed based on sequencing of seven “housekeeping” chromosomal genes (*abcZ, adk, aroE, fumC, gdh, pdhC*, and *pgm*) according to the protocol [7]. Allele numbers and sequencing types were assigned by comparison with GeneBank and PubMLST databases (http://www.ncbi.nlm.nih.gov/BLAST; https://pubmlst.org/; accessed on 30 September 2022).

The comparative study also included data on the genome of the reference strain FA19, as well as full genome sequences of 147 *N. gonorrhoeae* strains from various countries for phylogenetic analysis. WGS data were taken from the NCBI GenBank and PubMLST databases (Table S1).

### 2.2. Genome Sequencing

DNA was isolated using the Monarch Genomic DNA Purification Kit (New England Biolabs, Hitchin, UK). DNA libraries were prepared by DNA fragmentation followed by PCR indexing according to the Nextera XT DNA Library protocol (Illumina). After purification of the libraries, their size and concentration were checked using a TapeStation 4150 automated capillary electrophoresis platform (Agilent, Santa Clara, CA, USA). Full genomic sequencing of Russian strains was performed on a MiSeq system (Illumina, San Diego, CA, USA) using the MiSeq Reagent Kit V2 500 cycles (Illumina, San Diego, CA, USA). Nextera adapter read sequences were deleted using the trimmomatic v0.39 application.

### 2.3. Genome Assembly

The FastQC program (Babraham Institute, Cambridge, UK) was used to assess the quality of the sequencing performed; quality filtering was performed in the trimmomatic v 0.39 program with the parameters phred33, slidingwindow: 4:20, leading: 30, and trailing: 30. Genome coverage was normalized up to 40–60 in the program bbnorm (DOE Joint Genome Institute, Berkeley, CA, USA). Genomes were assembled de novo in the program SPAdes v3.13.1 (Saint Petersburg State University, St. Petersburg, Russia).

### 2.4. Bioinformatics Analysis

To search for the genetic causes of biochemical atypia, the genomes of Russian *N. gonorrhoeae* strains with normal and atypical metabolism were aligned with the reference annotated strain FA19 in the Mauve program 2015-02-26. BLAST service and the Ugene 41 program were used to establish the prevalence of found mutant gene variants among all deposited *N. gonorrhoeae* genomes.

To perform phylogenetic analysis of the studied sample of strains using single nucleotide substitutions of the core part of the genome, we used the Parsnp 1.6 program on Ubuntu 16. This method has high resolving power and allows us to identify characteristic genetic profiles of particular intraspecific groups and assess the degree of genetic variability of the species. The gingr 1.3 program was used to subsequently visualize the obtained analysis results with the construction of a heat map of mutation distribution in the genomes of the strains under study. Phylogenetic trees were visualized using Figtree 1.4 and Dendroscope 3.6.0.

## 3. Results

### 3.1. General Characterization of Genomes of Russian Clinical Isolates of G807 NG-MAST GENOGROUP/ST1594 MLST

Sixteen pre-selected Russian clinical isolates of *N. gonorrhoeae* belonging to the G807 NG-MAST GENOGROUP/ST1594 MLST were included in the study (Table 1). Eight of them exhibited typical biochemical properties (99% identification probability), and eight of them were found to have biochemical atypia, consisting in impaired amino acid metabolism and loss of D-glucose fermentation, leading to their misidentification as *Moraxella catarrhalis* or *Neisseria sicca* or a reduced probability of identification as *N. gonorrhoeae*.

All strains were additionally verified by mass spectrometry on a MALDI Microflex ionization time-of-flight mass spectrometer (Bruker Daltonics GmbH, Germany) with an identification index above 2.0 according to the manufacturers recommendations.

Russian clinical isolates of G807 NG-MAST genogroup/ST1594 MLST are characterized by sensitivity to six antibacterial drugs (penicillin, tetracycline, ciprofloxacin, ceftriaxone, spectinomycin, and azithromycin), which are analyzed under the RU-GASP. All of them were found to be sensitive to these antibacterial drugs

In the present study, we obtained the full genome sequences of 16 *N. gonorrhoeae* strains belonging to the G807 NG-MAST GENOGROUP/ST1594 MLST (Table 2). We deposited the obtained sequences into the NCBI GenBank database; the accession numbers are also listed in Table 2. All full genome sequences of the Russian strains under study have typical genome length, GC composition, and number of coding sequences (CDSs) for *N. gonorrhoeae* compared with the reference strain FA19.

**Figure 1:**
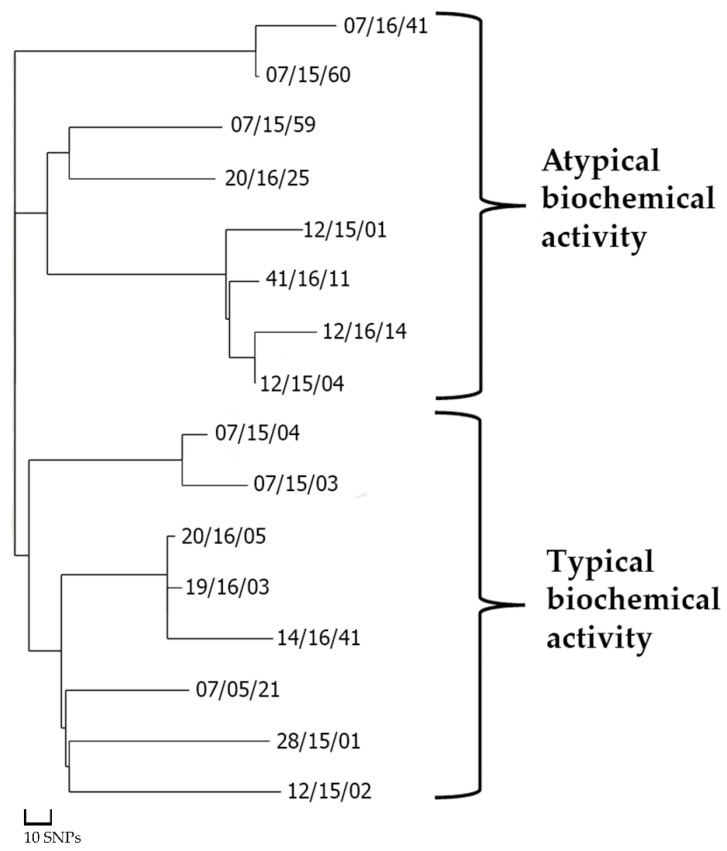
Clustering of Russian strains of *N. gonorrhoeae* G807 NG-MAST GENOGROUP/ST1594 MLST based on core single nucleotide substitutions.

During the primary analysis, we performed clustering using the analysis of single nucleotide substitutions of the core part of the genome. As a result, the resulting dendrogram clearly distinguished two clusters corresponding to the biochemical phenotype of their constituent strains (Figure 1).

This convinced us of the existence of genetic differences between typical and atypical strains and formed the basis for targeted analysis of nucleotide sequences of genes involved in the metabolism of amino acids and D-glucose.

### 3.2. Identification of Genetic Aspects of Biochemical Atypia

To identify the genetic causes of biochemical atypia, we performed a simultaneous alignment of all 16 full genome sequences of Russian gonococcal strains to the annotated FA19 reference genome in the Mauve 2015-02-26 program.

The clusters were distinguished by five single nucleotide substitutions of the core part of the genome, of which four are nonsynonymous (Table 3).

*DapC*

This gene is involved in the biosynthesis of the amino acid lysine, which is not counted among the taxonomically significant biochemical activities of *N. gonorrhoeae*.

*VT05_RS07040*

This gene is nonfunctional in *N. gonorrhoeae* because of a reading frame shift and is not involved in cell metabolism.

*rpsE*

This gene encodes the S5 protein of the small ribosomal subunit. Mutations in this protein can lead to resistance to spectinomycin, such as Lys-28 → Glu, Thr-24 → Pro substitutions, deletion of codon 27 (valine) [8,9]. However, the mutation we described does not affect the functional sites of this protein and does not change the sensitivity.

*VT05_RS09720*

This gene encodes an integral protein that ensures the transport of Na^+^ and H^+^ ions across the cell membrane.

*Nth*

This gene encodes an endonuclease involved in the DNA repair process, the release of damaged pyrimidines from the DNA chain.

Thus, the detected genetic differences did not correlate with the taxonomically significant biochemical activities of *N. gonorrhoeae* and had to be evaluated as a separate aspect of their genetic evolution.

**Figure 2:**
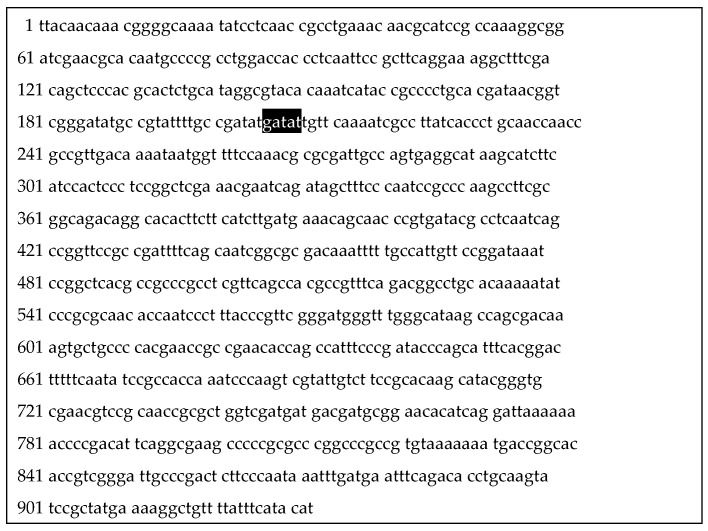
Localization of a 5 bp deletion at positions 733–737 in the *pip* gene of Russian atypical strains of *N. gonorrhoeae*; the gene sequence is provided in reverse complementary form.

In this context, the analysis of the genetic causes of atypical amino acid metabolism and glucose assimilation included the sequences of genes whose products have proven involvement in amino acid and glucose metabolism. In the first case, the sequences of genes were involved in the aminopeptidase activity of *N. gonorrhoeae*. According to the modern enzyme classification, L-prolarylamidase (ProA) is now called prolyl iminopeptidase and is encoded by the *pip* gene. In all atypical Russian strains, a 5-nucleotide insertion (ATATC) at positions 733–737 from the beginning of the gene was detected (Figure 2). This mutation leads to the formation of a stop codon with premature termination of enzyme synthesis and the formation of a defective product with a length of 244 amino acid residues (the length of the full enzyme is 310 amino acid residues).

The absence of glucose assimilation was observed in five of the strains we studied. To search for mutations affecting glucose assimilation, we analyzed the sequences of *pgl*, *eda*, *pgi*, *gnd*, *edd*, *tpiA*, and *zwf* genes (encoding the enzymes 6-phosphogluconolactonase, 2-dehydro-3-deoxy-phosphogluconate aldolase, glucose-6-phosphate isomerase 1, 6-phosphogluconate dehydrogenase, phosphogluconate dehydratase, triosephosphate isomerase, and glucose-6-phosphate 1-dehydrogenase necessary for glucose assimilation [10]. The analysis was performed using gingr 1.3 and MAUVE 2015 software in all 16 Russian strains compared with the genome of the wild-type FA19 strain. As a result, we found a large number of nonsynonymous SNPs in the *tpiA*, *pgi*, *zwf*, *gnd*, *edd*, and *eda* genes. However, almost all the identified nucleotide substitutions were present in both strains with atypia and strains with normal glucose assimilation. As a result, only one mutation was found exclusively in the cluster of strains with atypia, in the *pgi* gene encoding glucose-6-phosphate isomerase. This mutation is present only in 2 strains, 07/15/60 and 07/16/41. The nucleotide substitution G → T is localized in position 274 from the beginning of the gene and results in a substitution of alanine for serine. However, the significance of this mutation in impaired glucose assimilation cannot yet be unequivocally proven and requires additional studies. It should be noted that *N. gonorrhoeae* has two *pgi* genes in its chromosome; the found mutation is located in only one gene.

### 3.3. Phylogenetic Analysis of the G807 NG-MAST GENOGROUP/ST1594 MLST in the Global Population of N. gonorrhoeae

A comparative analysis of 16 sequenced and 147 known genomes in the global population of *N. gonorrhoeae* was performed to understand the prevalence of the detected genetic features leading to biochemical atypia. Strains from the United States, the European Union, Asia, etc., whose full genome nucleotide sequences were taken from the NCBI and PubMLST databases, were used in the construction of the phylogenetic tree.

**Figure 3:**
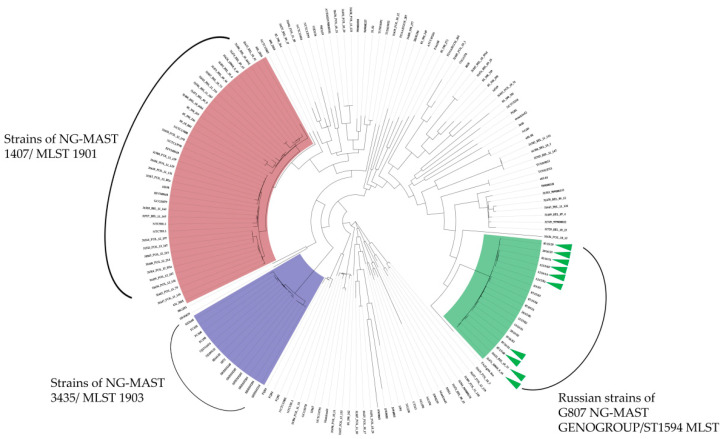
Phylogenetic analysis of the global diversity of *N. gonorrhoeae* was performed using the Maximum likelihood algorithm in Parsnp 1.6. Green triangles mark the Russian strains of the G807 NG-MAST GENOGROUP/ST1594 MLST with biochemical atypia, the cluster of strains from Asian countries are represented by the clones of strain FC428 of the NG-MAST 3435/MLST 1903 and strains of the NG-MAST 1407/MLST 1901 isolated in European countries.

Using the Parsnp 1.6. program, we identified 20,038 core single nucleotide substitutions (SNPs) that make up the genetic profiles of the strains we studied. This parameter with a relatively small genome size of *N. gonorrhoeae* genome size of 2.1 million bp indicates a high degree of genetic heterogeneity of this bacterial species. The obtained genetic profiles of strains were used to construct a phylogenetic tree using the Maximum likelihood algorithm and the GTR substitution model (Figure 3).

The resulting phylogenetic tree reflects the high internal genetic variability of the *N. gonorrhoeae*, all major clusters of strains are located on branches extending to hundreds of SNPs.

The first cluster is represented mainly by strains isolated in Asian countries, such as Japan and China, most of which are clones of the Japanese strain FC428 first isolated in Japan in 2015, and which have increased resistance to the current drugs of choice for the therapy of gonococcal infection, ceftriaxone and azithromycin [11].

The second cluster is formed mainly by strains from Belarus and Poland, as well as from other European countries belonging to the NG-MAST 1407/MLST 1901.

Russian strains formed a separate cluster on the phylogenetic tree; it included all 16 strains studied and two strains isolated in Belarus (Figure 3), which also belonged to the G807 NG-MAST GENOGROUP/ST1594. At the same time, the two previously described isolates from Belarus were also found to have five nucleotide insertions in the *pip* gene and a nonsynonymous nucleotide substitution in the *pgi* gene, which are characteristic of modern Russian isolates with atypical biochemical activity.

Overall, the G807 NG-MAST GENOGROUP/ST1594 cluster has 170 unique nucleotide substitutions, of which 94 are nonsynonymous and result in either an amino acid substitution or a stop codon. The entire cluster is characterized by a large number of SNPs in the *fabD* gene involved in fatty acid biosynthesis: 21, of which 6 are nonsynonymous. At the same time, widely known mutations in the *penA, ponA, porB, mtrR*, etc., genes, determining multiple antimicrobial resistance were absent in the genomes of G807 NG-MAST GENOGROUP/ST1594 (the only mutation identified was Asp345a Insertion in Penicillin-Binding Protein 2), which indicates the specificity of the conservation strategy of this genogroup within the global population of *N. gonorrhoeae*, different from that in NG-MAST 3435/MLST 1903 and NG-MAST 1407/NG-MLST 1901 [12].

## 4. Discussion

An important feature of the *N. gonorrhoeae* is a high level of genotypic and subsequent phenotypic variability manifested in the emergence of new molecular subtypes, the spread of antimicrobial resistance determinants and virulence factor variations [13,14]. Another aspect of such variability is the reduction in the number of biochemical activities leading to the development of autoxotrophy in some cases accompanied by the loss of taxonomically significant characteristics with incorrect identification of such clinical isolates [15].

The problem of *N. gonorrhoeae* biochemical atypia is currently relevant in Russia, which manifests itself in the impossibility of correct identification of clinical isolates analyzed under the RU-GASP program. At the same time, the significance of this is determined by the belonging of atypical strains to the G807 NG-MAST GENOGROUP/ST1594 that have been dominant in Russia in recent years [3,4].

To investigate the nature of this phenomenon, we formed a group of 16 clinical isolates belonging to the G807 NG-MAST GENOGROUP/ST1594 which showed typical (8 isolates) or atypical (8 isolates) biochemical activity profiles using NH cards on a VITEK 2 Compact analyzer (BioMérieux, France). Whole genome sequencing of these isolates showed a clear correspondence between typical and atypical variants in two different clusters, which confirmed the working hypothesis about the genetic nature of the analyzed phenomenon.

The search for direct causes of biochemical atypia led us to the discovery of a 5-nucleotide insertion (ATATC) in the *pip* gene, which interrupted the protein chain synthesis of an enzyme commonly estimated to be L-prolarylamidase (prolyl iminopeptidase). This insertion was first detected in two strains isolated in Sweden in 2000, belonging to the NG-MAST 3369 sequencing type, which is also a member of the G807 NG-MAST GENOGROUP/ST1594 MLST (C → T substitution at position 356 of the *porB* gene) [16]. In addition, an identical insertion was detected in the genomes of two clinical isolates of *N. gonorrhoeae* from the Republic of Belarus, also belonging to the G807 NG-MAST GENOGROUP/ST1594 MLST [17]. There is no information about its presence in modern NG-MAST types or the extent of its distribution in the global population in the available literature. Such mutations are quite rare in the *N. gonorrhoeae*, whose mutational variability is usually associated with single nucleotide mutations, but as the present study shows, this insertion was most common in the Russian population of *N. gonorrhoeae*.

Typically, the dysfunction of the *pip* gene is associated with a single nucleotide deletion of thymidine at position 110, resulting in a frameshift and synthesis of a 123 amino acid residue defective protein, and with a thymidine insertion at position 157, also resulting in synthesis of a 55 amino acid residue defective protein. All this makes it difficult or impossible to diagnose the caused disease using the prolyl iminopeptidase rapid test and makes detection into several commercial test panels difficult. Clinical isolates with such mutations often belong to NG-MAST 210, 292, and 1259 [16,17,18,19].

Thus, the results of this study indicate the unity of origin of biochemically atypical strains of *N. gonorrhoeae*, the rather rare insertion of five nucleotides in the *pip* gene being inherited exclusively vertically and being spread only within a certain genogroup.

Discussing the evolutionary reasons for the loss of biochemical features, we can assume that the general trend in the molecular evolution of *N. gonorrhoeae*, as well as other pathogens, is reduced to the functional and structural reduction in genome elements, due to their energy inexpediency in the functioning of the bacterial cell, which is an obligate parasite [20].

In turn, the practical aspect of this observation is the need to phase out the routine biochemical identification of *N. gonorrhoeae* with a transition to MALDI-TOF MS technologies when conducting RU-GASP providing additional analytical capabilities [21,22].

Another important observation is the comparative phylogenetic analysis of the G807 NG-MAST GENOGROUP/ST1594 in the global population of *N. gonorrhoeae*. It was established that the G807 NG-MAST GENOGROUP/ST1594 cluster represented predominantly by the Russian strains was considerably distant from the clusters of the NG-MAST 1407/MLST 1901 and NG-MAST 3435/MLST 1903 distributed abroad with multiple genetic determinants of resistance to antimicrobial drugs [23,24].

Resistance to ceftriaxone in the cluster of NG-MAST 3435/MLST 1903 is explained by the presence of a mutant variant of the gene allele penA-60.001, which contains two key genetic determinants, A311V and T483S, as well as G545S, I312M, and V316T [11]. This clone was subsequently reported in 2017 in Australia (A7536, A7846), Canada (47707), Denmark (GK124) and France (F90), and in 2018 in Ireland (IR72). The recent emergence of increasingly resistant members of this group, including recent cases of gonorrhea in Cambodia, is of high concern to international public health [23,25,26].

The cluster of NG-MAST 1407/MLST 1901 includes isolates with multiresistance to antibiotics and has molecular mechanisms contributing to disruption of AMP entry, their increased efflux, as well as modification of target proteins. At the same time, the modern selection of representatives of the named genogroups NG-MAST and clonal complex NG-STAR in the global population is associated precisely with their high level of antimicrobial resistance [24,27,28].

At the same time, the absence of similar determinants in the G807 NG-MAST GENOGROUP/ST1594 genomes suggests a different strategy for their persistence in the global *N. gonorrhoeae* population, consisting not in resistance to antimicrobial therapy, but in treatment avoidance as a result of erroneous laboratory diagnosis.

## 5. Conclusions

The loss of L-prolarylamidase (ProA) enzyme activity determined by a 5-nucleotide insertion in the *pip* gene. This mutation is currently detected in Russian strains of G807 NG-MAST GENOGROUP/ST1594 and two strains isolated in Belarus belonging to the same genogroup. The hypothesis of structural and functional reduction in the gonococcal genome as an intracellular pathogen is among the possible reasons for the loss of biochemical activity in a number of features. A phylogenetic study of Russian strains of *N. gonorrhoeae* belonging to the G807 NG-MAST GENOGROUP/ST1594, currently dominant in the Russian Federation, was carried out. The cluster of Russian strains is at a considerable genetic distance from strains of such common genogroups as NG-MAST 3435/MLST 1903 and NG-MAST 1407/MLST 1901 and has a large number of unique mutations.

## Figures and Tables

**Table 1:** Russian clinical isolates of *N. gonorrhoeae* included in the study.

Number in the SSCDC * Collection	Source of Obtaining (Isolation Site, Sex, Age, Region)	Biochemical Activity Test Results	NG-MAST and MLST Type
07/15/03	ECS **; W, 25, Arkhangelsk region	TBA ***; 99% *Neisseria gonorrhoeae*	807/1594
07/15/04	US; M, 18, Arkhangelsk region	TBA, 99% *Neisseria gonorrhoeae*	12,529 (*tbpB* C → T in the position 381)
07/15/59	US; M, 34, Arkhangelsk region	ABA (defective amino acids and glucose metabolism); 94% *Neisseria cinerea* ****	1531 (*porB* G → A in the position 127)
07/15/60	US; M, 26, Arkhangelsk region	ABA (defective amino acids and glucose metabolism); 95% *Moraxella catharrhalis* ****	807
07/16/41	US; M, 22, Arkhangelsk region	ABA (defective amino acids and glucose metabolism); 97% *Moraxella catharrhalis* ****	807
12/15/01	US; M, 29, Chuvash Republic	ABA (defective amino acids metabolism); Low discrimination *Moraxella catharrhalis/Neisseria gonorrhoeae* ****	5941 (*porB* A → G in the position 341)
12/15/02	US; M, 26, Chuvash Republic	TBA, 99% *Neisseria gonorrhoeae*	807
12/15/04	US; M, 43, Chuvash Republic	ABA (defective amino acids and glucose metabolism); Low discrimination *Moraxella catharrhalis/Neisseria cinerea* ****	807
12/16/14	US; M, 25, Chuvash Republic	ABA (defective glucose metabolism); 96% *Neisseria gonorrhoeae*	807
14/16/41	US; M, 41, Arkhangelsk region	TBA, 99% *Neisseria gonorrhoeae*	807
19/16/03	ECS; W, 23, Omsk region	TBA, 99% *Neisseria gonorrhoeae*	807
20/16/05	US; W, 31, Kaluga Region	TBA, 99% *Neisseria gonorrhoeae*	807
20/16/25	US; M, 52, Kaluga Region	ABA (defective amino acids metabolism); 92% *Neisseria gonorrhoeae*	9576 (*porB* T → G in the position 349)
28/15/01	US; M, 25, Penza region	TBA, 99% *Neisseria gonorrhoeae*	9570 (*porB* G → A in the position 340)
41/16/11	US; M, 30, Tomsk region	ABA (defective amino acids metabolism); Low discrimination *Moraxella catharrhalis/Neisseria gonorrhoeae//Neisseria meningitidis* ****	807
07/05/21	Arkhangelsk region	TBA, 99% *Neisseria gonorrhoeae*	807

* SSCDC—State scientific center of dermatovenerology and cosmetology; ** ECS—endocervical specimen; US—urethral swab; *** TBA—typical biochemical activity; ABA—atypical biochemical activity; ****—misidentification improved by MALDI Microflex analyses.

**Table 2:** General characterization of the genomes of Russian strains of *Neisseria gonorrhoeae* G807 NG-MAST GENOGROUP/ST1594 MLST and the reference strain FA19 (USA).

Sample	Contigs	Length of Contigs. mb	N50 of Contigs	GC of Contigs (%)	Genes	CDS	ncRNA	rRNA	tRNA	tmRNA	Accession
07/15/03	135	2.3	43.5	52.5	2165	2095	16	3	50	1	JANQDD000000000
07/15/04	99	2.3	53.1	52.5	2210	2140	16	3	50	1	JANUFX000000000
07/15/59	93	2.3	67.6	52.5	2208	2141	16	3	47	1	JANUFY000000000
07/15/60	110	2.3	48.5	52.5	2211	2140	17	3	50	1	JANUFZ000000000
07/16/41	124	2.3	48.3	52.5	2210	2139	17	3	50	1	JANUGA000000000
12/15/01	98	2.2	53.3	52.5	2159	2090	15	3	50	1	JANUGB000000000
12/15/02	96	2.2	55.3	52.5	2164	2095	15	3	50	1	JANUGC000000000
12/15/04	77	2.2	67.2	52.5	2163	2094	16	3	49	1	JANUGD000000000
12/16/14	122	2.3	52.4	52.4	2162	2093	16	3	49	1	JANUGE000000000
14/16/41	113	2.2	53.6	52.4	2160	2091	15	3	50	1	JANUGF000000000
19/16/03	99	2.2	59.0	52.5	2164	2095	15	3	50	1	JANUGG000000000
20/16/05	116	2.2	53.2	52.5	2169	2100	15	3	50	1	JANUGH000000000
20/16/25	123	2.3	50.1	52.4	2167	2097	16	3	50	1	JANUGI000000000
28/15/01	111	2.2	53.4	52.4	2165	2096	15	3	50	1	JANUGJ000000000
41/16/11	97	2.2	48.7	52.5	2157	2088	15	3	50	1	JANUGK000000000
07/05/21	116	2.3	48.3	52.4	2166	2097	15	3	50	1	JANUGL000000000
FA19	1	2.2	-	52.4	2332	2261	4	3	55	N/A	NZ_CP012026

**Table 3:** Five unique SNPs discriminating between *N. gonorrhoeae* clades with typical and atypical biochemical activity.

Gene	Product	SNP Position in the FA19 Genome	Type of Substitution	Synonymity of Substitution
*DapC*	succinyldiaminopimelate transaminase	193,614	G → A	Nonsyn (Glu→Lys)
VT05_RS07040	galactose mutarotase—pseudogen (frameshifted)	1,331,807	C → T	-
*rpsE*	30S ribosomal protein S5	1,607,962	G → A	Nonsyn (Ala→Ser)
VT05_RS09720	Na^+^/H^+^ antiporter family protein	1,800,365	C → T	Syn
*nth*	endonuclease III	2,119,191	C → T	Nonsyn/STOP codon

## Data Availability

All new WGS data of *N. gonorrhoeae* strains obtained as part of this study are deposited in the NCBI GenBank with the accession numbers shown in Table 2. All *N. gonorrhoeae* strains used in the phylogenetic analysis are listed in the Table S1.

## Supplementary Materials

The following supporting information can be downloaded at: https://www.mdpi.com/article/10.3390/microorganisms10112271/s1, Table S1: Genomes used for phylogenetic analysis of Neisseria gonorrhoeae.
